# Antifungal Minimal Inhibitory Concentrations of Mold Isolates from Patients with Cancer; Single-Center Experience, 2018–2023

**DOI:** 10.3390/jof11070518

**Published:** 2025-07-12

**Authors:** Hafij Al Mahmud, Sanjeet Singh Dadwal, Rosemary C. She

**Affiliations:** 1 Department of Pathology, City of Hope National Medical Center, Duarte, CA 91010, USA; 2 Department of Medicine, Division of Infectious Disease, City of Hope National Medical Center, Duarte, CA 91010, USA

**Keywords:** antifungal susceptibility, epidemiological cut-off values, *Aspergillus* spp., immunocompromised

## Abstract

The increasing emergence of antifungal resistance poses potential clinical challenges, particularly among immunocompromised patients with cancer at risk of invasive mold infections, but data on antifungal susceptibility trends specific to this population are few. We evaluated distributions of minimal inhibitory concentrations (MIC), including minimal effective concentrations (MEC) for echinocandins, of 11 antifungal agents for 523 mold isolates (395 *Aspergillus* spp.) from cancer patients. Based on published Clinical and Laboratory Standards Institute guidelines, isavuconazole had notably high rates of non-wild-type MICs for *A. fumigatus* (19.6%), *A. flavus/oryzae* (34.8%), *A. niger* complex (26.1%), and *A. terreus* complex (8.33%). Persistent low baseline resistance of *A. fumigatus* to voriconazole was observed across multiple years (2.4–11.5% per year, average 8.41%) without significant trends in MIC change over time. Itraconazole and posaconazole demonstrated the lowest MIC distributions (MIC_50_ ≤ 0.06–0.5 µg/mL) of the azoles against *Aspergillus* spp. Amongst the *A. niger* complex, 29.4% (27/92) demonstrated non-wild-type MICs to itraconazole. While the *A. nidulans* group was less frequent (*n* = 24), bimodal peaks in MIC/MEC were noted for caspofungin (≤0.06 and 1 µg/mL). Non-*Aspergillus* molds of significance (Zygomycetes, *Fusarium* spp., *Scedosporium* spp., and *Lomentospora prolificans*) demonstrated variable but increased MICs to antifungal agents as previously described. Our results highlight increased rates of non-wild type MICs for *Aspergillus* spp. to isavuconazole and voriconazole, which are commonly used antifungal agents in cancer patients. Such AST trends should be closely monitored in populations with frequent antifungal use and encourage increased antifungal stewardship efforts.

## 1. Introduction

Invasive mold infections (IMIs) are increasingly recognized in patients with immune compromise or severe underlying conditions [1]. Out of an estimated 6.5 million cases of invasive fungal infections globally each year, approximately 2.1 million individuals develop invasive aspergillosis (IA) among patients with different underlying conditions including cancer [2]. An estimated 14,820 hospitalizations occurred due to *Aspergillosis* infection in 2014 in the USA [3]. IA is a serious opportunistic infection encountered following intensive chemotherapy for hematologic malignancies or hematopoietic stem cell transplantation [4]. Despite advances in antifungal therapies, IA in intensive care unit patients remains associated with high mortality rates, with crude mortality exceeding 80% and attributable mortality ranging between 18 and 48% [5]. The genetic adaptability of molds, limited antifungal agents available, breakthrough infection on anti-mold prophylaxis, and incomplete antimicrobial susceptibility testing (AST) standards and clinical breakpoint developments pose challenges in defining the optimal treatment for IMIs [6,7].

When clinical breakpoints are limited, epidemiological cutoff values (ECOFFs/ECVs) may help in monitoring local susceptibility trends to inform empirical treatment decisions [8]. The ECV values, defined as the upper limit of the wild-type population (wt-UL), marks the highest minimal inhibitory concentration (MIC) of the wild-type population, ensuring that the isolates used for its determination lack resistance mechanisms [9,10].

Isavuconazole, a triazole antifungal, is widely used for the treatment of invasive aspergillosis and other clinically important fungal infection caused by molds and yeasts [11,12]. Voriconazole is another potent antifungal compound used as a first line defense against invasive aspergillosis [13]. Posaconazole and amphotericin or liposomal amphotericin B are also used to treat infections caused by *Aspergillus* spp. and Mucorales [14,15]. Echinocandins (e.g., caspofungin, anidulafungin, and micafungin), despite their limited efficacy against *Aspergillus* spp., serve as an alternative option for treating invasive aspergillosis due to their favorable tolerability and ability to enhance the effects of other antifungal agents and utility in salvage therapy [14,16]. To date, there are few studies examining the trends in MIC and non-wild-type (NWT) populations in cancer patient populations receiving antifungal prophylaxis, empiric therapy, or directed therapy. The purpose of this study is to present the MIC distributions of commonly used antifungal agents for clinically relevant molds isolated from an immunocompromised cancer patient population who often receive triazole antifungal prophylaxis. We compare our data to published MIC distributions and ECVs to understand antifungal MIC trends in a high-risk patient population.

## 2. Materials and Methods

### 2.1. Mold Isolates and Analysis of Minimal Inhibitory Concentrations

This was a retrospective study of mold isolates obtained from fungal cultures performed as the standard of care, 2018–2023 (City of Hope Comprehensive Cancer Center, Duarte, CA, USA). Isolates were routinely identified by mass spectrometry (Vitek MS, Biomerieux, Durham, NC, USA), with morphologic assessment and rDNA gene sequencing used as secondary methods as needed. First mold isolated per patient source per encounter were routinely submitted as the standard of care for AST (ARUP Reference Laboratories, Salt Lake City, UT, USA) following the CLSI-recommended broth microdilution method using RPMI 1640 medium [17]. Per CLSI standard, AST tests were performed once per isolate following validated methods under strict quality control. Reported MIC and MEC (minimum effective concentration) data were collected and de-identified prior to analysis. The standard AST panel included flucytosine (5-FC), amphotericin B, fluconazole, isavuconazole, itraconazole, posaconazole, voriconazole, ketoconazole, micafungin, anidulafungin, and caspofungin. The concentration ranges tested were as follows: flucytosine (0.06–64 µg/mL), amphotericin B (0.12–8 µg/mL), anidulafungin, caspofungin, and micafungin (0.06–8 µg/mL), and isavuconazole, itraconazole, ketoconazole, posaconazole, and voriconazole (0.06–16 µg/mL).

MIC_50_, MIC_90_, and modal MICs were calculated for each antifungal–organism pair. MIC_50_ and MIC_90_ refer to the MIC values representing the 50th and 90th percentile MIC for each antifungal–organism agent combination, respectively. Additionally, MEC data were similarly analyzed for echinocandins and refer to the lowest concentration of antifungal agent that results in small, rounded compact hyphal forms. MEC_50_, MEC_90_, and modal MECs were calculated for each echinocandins antifungal–organism pair. Statistical calculations were performed in R (version 4.4.2) with the dplyr package (version 1.1.4). For the clinical context, population level-cancer diagnoses were extracted over the study time period and sorted by mold group, but not at the individual patient or isolate level due to the de-identification of the AST dataset. The study protocol was determined by the Institutional Review Board at City of Hope not to be human subjects research.

### 2.2. Determining ECVs and Wild-Type Upper Limit (wt-UL)

For the purposes of comparisons to published ECVs, we determined the ECVs for our dataset using the ECOFFinder algorithm following standard methods [18]. Briefly, cumulative numbers of MIC distribution data collected from organism–agent combinations were modeled using non-linear regression to fit a cumulative log-normal distribution [18]. ECOFFinder is best-suited for unimodal, normally distributed data that closely fit the distribution curve; otherwise, it may fail to determine ECVs, as we observed in many of the tested combinations. The wt-UL was defined as two MIC steps higher than the modal MIC [19]. Even though both ECVs and wt-UL define the upper limit of the MIC value that differentiates wild type isolates from NWT isolates, we designated the latter as wt-UL to distinguish it from ECOFFinder-generated ECVs.

## 3. Results

### 3.1. Distribution of Isolated Mold Pathogens 

Overall, 1025 clinical fungal isolates from 784 cancer patients were submitted for fungal AST (Table S1, Figure S1). The most prevalent molds were *Aspergillus fumigatus* (*n* = 215), *A. niger* complex (*n* = 96), Zygomycetes (*n* = 49), *Fusarium* spp. (*n* = 27), *Scedosporium apiospermum/S. boydii* (*n* = 22), and *Lomentospora prolificans* (*n* = 17). According to Poisson regression analysis, there was a statistically significant upward trend in the observed counts of *Aspergillus* spp. over time from 2018 to 2023 (*p* < 0.001).

Most *Aspergillus* isolates were from the lower respiratory tract (376/412, 91.3%), as were Zygomycetes (40 respiratory, 1 blood, 8 tissue) and *S. apiospermum/S. boydii* (18 respiratory, 3 blood, 1 tissue). By contrast, *Fusarium* spp. were most-isolated from blood (13 blood, 8 tissue, 5 respiratory tract), as was *L. prolificans* (10 blood, 4 respiratory tract, 3 tissue). Additionally, the overall distribution of specimen sources of the mold isolates is presented in Figure S2. Briefly, respiratory samples contributed the highest proportion (81.16%) of total mold isolates (*n* = 605), followed by tissue (7.77%), blood (5.12%), wound (1.65%), body fluid (1.49%), bone, urine, bone marrow, cerebrospinal fluid, ear, skin, and other sources (0.17–0.83%).

Cancer diagnoses associated with *Aspergillus* spp. recovered in cultures were more balanced between solid organ malignancies (45.9%) and hematologic malignancies (51.7%), compared to *Fusarium* spp., which were only isolated from patients with hematologic malignancies (20 leukemia, 2 myelodysplastic syndrome, 4 lymphoma). *Scedosporium/Lomentospora* spp. and Mucorales were likewise predominantly associated with hematologic malignancies (87.5% and 85.2%, respectively) rather than solid organ malignancies (14.2% and 11.1%, respectively), with a small number of combined hematologic and solid organ malignancy cases seen for Mucorales (3.7%).

### 3.2. Distribution and Trends in MICs

**Figure 1:**
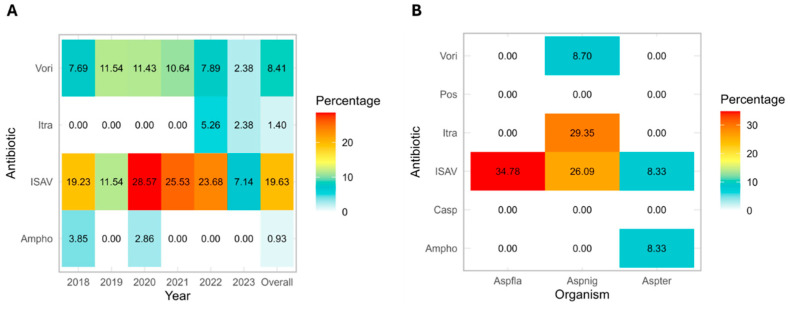
Emergence of non-wild-type (NWT) *Aspergillus* spp. against different antifungal agents. NWT to voriconazole (Vori), itraconazole (Itra), isavuconazole (ISAV), and amphotericin B (Ampho) is displayed as percentages, for *A. fumigatus* over the years (**A**), and over the entire study period for *A. flavus, A. niger*, and *A. terreus* (**B**).

The MIC/MEC_50_, MIC/MEC_90_, mode, range, and ECVs for each mold organism–drug combination were tabulated (Table 1, Table 2 and Table 3). For *Aspergillus* spp., *A. terreus* complex predictably demonstrated elevated MIC_50_ (2 µg/mL) and range (0.5 to ≥8 µg/mL) for amphotericin B. The other *Aspergillus* species had overall non-wild-type (NWT) MIC rates for amphotericin B of <1.0% (Figure 1). MIC/MEC_50_ values for echinocandins were uniformly low (≤0.06 µg/mL) except for caspofungin with *A. nidulans* (MIC/MEC_50_: 0.5 µg/mL), which demonstrated an unexpected bimodal pattern, with one mode of ≤0.06 µg/mL and a second mode of 1 µg/mL (Table 3). Among mold active azoles, itraconazole and posaconazole demonstrated the lowest MIC distributions (MIC_50_ ≤ 0.06–0.5 µg/mL) compared to isavuconazole and voriconazole (MIC_50_ 0.25–4 µg/mL) for *Aspergillus* spp. Specifically, *A. nidulans* and *A. terreus* complex showed the lowest MIC_50_ (≤0.06 µg/mL), whereas *A. niger* complex demonstrated the highest MIC_50_ (0.5 µg/mL). For isavuconazole, *A. niger* complex and *A. lentulus* had higher MIC_50_ (4 µg/mL) than other *Aspergillus* species (0.25–1 µg/mL). *A. lentulus*, while consisting of only nine isolates, demonstrated relatively high MICs to amphotericin B (MIC_50_ of 4 µg/mL) and azole agents except for posaconazole (range ≤ 0.06–0.5 µg/mL).

**Figure 2:**
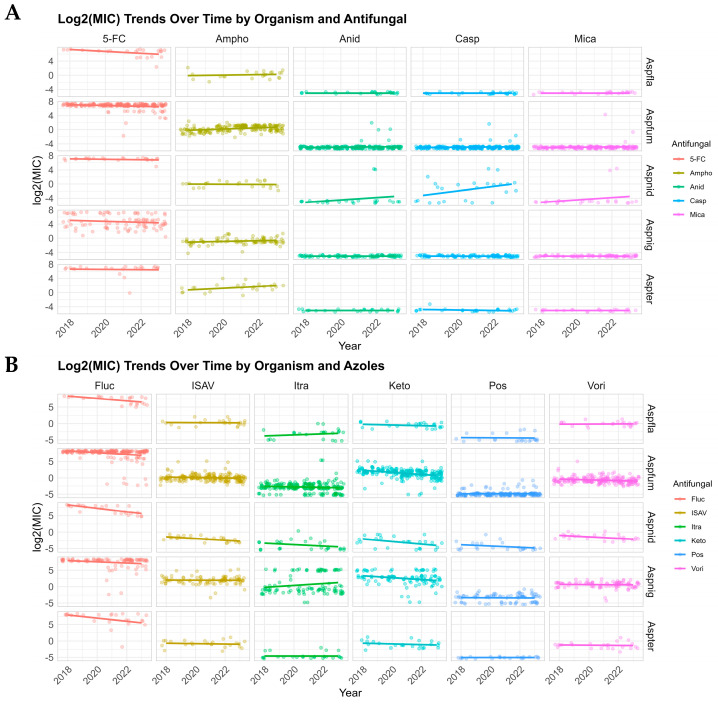
Overall MIC trends of different antifungal compounds against *Aspergillus* spp. by year, 2018–2023. The data were log_2_-transformed and then linear regression models (log_2_MIC ~ year) were fitted for each organism–antifungal ((**A**); azoles, (**B**); other antifungals) combination to estimate temporal trends. Complementary non-parametric Mann–Kendall tests were performed to assess monotonic trends without assuming linearity. Although visual increases in amphotericin B MICs against *A. fumigatus*, *A. niger*, and *A. terreus*, increases in echinocandin MICs against *A. nidulans*, and decreases in MICs for fluconazole and ketoconazole against all *Aspergillus* spp. are apparent, none were statistically significant (*p* > 0.05).

Changes in antifungal MICs for *Aspergillus* species were evaluated over time (Figure 2A,B). Overall, we visually observed increasing amphotericin B MICs over time for *A. fumigatus*, *A. niger*, and *A. terreus* (Figure 2A). Anidulafungin, caspofungin, and micafungin showed rising MIC trends against *A. nidulans* (Figure 2A). Interestingly, ketoconazole exhibited decreasing MIC trends across all *Aspergillus* species (Figure 2B). Some antifungal MICs remained stable throughout the study period of 2018–2023. Although these trends were visually suggested (Figure 2A,B), no statistically significant changes were identified (*p* > 0.05).

For non-*Aspergillus* mold species, MIC distributions largely confirmed the expected patterns by group (Table 2), though no CLSI ECVs are available for comparison [20,21]. Antifungal agents showed little overall activity against *Fusarium* spp., though some variation was observed in the MIC range for voriconazole, posaconazole, and amphotericin B. *Scedosporium* spp., which generally showed elevated MICs to most agents including amphotericin B (MIC_50_ ≥ 8 µg/mL) with more variability for voriconazole and posaconazole. For Zygomycetes, posaconazole had the most effective MICs among the azole agents, followed by itraconazole and isavuconazole. Due to small numbers, individual species of Zygomycetes and *Fusarium* were not analyzed separately. Zygomycetes MICs for isavuconazole significantly correlated with posaconazole (Spearman’s ρ = 0.857, *p* < 0.001) and itraconazole (Spearman’s ρ = 0.842, *p* < 0.001) MIC values. Against Amphotericin B, 93.8% (46/49) of Zygomycetes MICs were ≤2 µg/mL.

### 3.3. Emergence of Non-Wild-Type Mold Isolates

Compared to published CLSI ECVs for species with >90 isolates (*A. fumigatus* and *A. niger* complex), most ECVs were similar but several key differences emerged. For both species, our calculated ECVs for isavuconazole and voriconazole were two-fold higher. Accordingly, isavuconazole NWT rates based on CLSI criteria were notably increased across *Aspergillus* spp.: *A. flavus/oryzae* (34.78%), *A. niger* complex (26.09%), *A. fumigatus* (19.63%), and *A. terreus* complex (8.33%). Against voriconazole, 8.41% of *A. fumigatus* were resistant, though rates varied by year (2.4–11.5%) (Figure 1A,B). The Voriconazole NWT rate was similar for *A. niger* complex (8.33%). Finally, our ECVs for itraconazole were two- to four-fold lower than the published CLSI ECVs for *A. fumigatus* and *A. niger* complex. However, the MIC distribution for *A. niger* complex was not unimodal, with 27 of 92 isolates (29.3%) demonstrating NWT MICs of ≥8 µg/mL.

## 4. Discussion

Our study identifies *Aspergillus* as the most frequently isolated fungal pathogen from our cancer center. The dominance of *Aspergillus* spp. aligns with the existing literature, as this genus is commonly associated with IMIs in immunocompromised cancer patients, exceeding the frequency of invasive candidiasis [22,23]. IA remains associated with high mortality rates in critically ill patients, with crude mortality exceeding 80% and attributable mortality ranging between 18 and 48% [5].

The antifungal AST data presented here largely reflect other published studies based on the CLSI broth microdilution method, except for notable MIC elevations to isavuconazole for *Aspergillus* spp. (8.3–34.8% NWT) and decreased in vitro activity for voriconazole against *Aspergillus* compared to other triazoles [20,24]. The SENTRY surveillance program found *A. fumigatus* NWT rates for isavuconazole of 3.8% (2015–16) to 6.0–7.3% (2017–21) [20,25], while our NWT rate of 20.0% was appreciably higher. Among studies focused on cancer patient populations, one retrospective cohort analysis identified *A. fumigatus* in one or multiple specimens from 13 cancer patients, with isolates from three of those patients exhibiting resistance to triazole antifungals [26]. Another study conducted at a tertiary care cancer center found that approximately 13% of *Aspergillus* spp. isolates collected from 1999 to 2015 were classified as NWT for triazoles [27]. Additional studies in patients with cancer have utilized other AST methods such as gradient diffusion strips and may not be directly comparable, but nonetheless suggest a wide range of resistance rates in *Aspergillus* species to azole agents, from <1% to nearly 30% [28,29].

While we identified a significant correlation between isavuconazole and voriconazole MIC values for *A. fumigatus* (Spearman’s ρ = 0.529, *p* < 0.001), this correlation was weaker than others have previously reported (Spearman’s ρ = 0.885–0.887) [30,31]. The reasons for this difference are unclear, but inter-laboratory technical variations in MIC determinations may contribute. In addition, adaptive responses in mold isolates that differentially affect MICs for voriconazole and isavuconazole may be possible given that the practice at our center favors the usage of isavuconazole for either antifungal prophylaxis or treatment, with very low voriconazole utilization due to drug–drug interactions and less predictable pharmacokinetic profiles. Our study also revealed a notably higher percentage (29.4%) of *A. niger* NWT isolates for itraconazole. This finding aligns with broader increased itraconazole resistance trends observed in the *A. niger* complex organisms. One large study using the gradient diffusion method and EUCAST interpretations found that over half (52%) of *A. niger* complex isolates were resistant to itraconazole, with rates varying across cryptic species from 33 to 100% [32]. Cross-resistance to other azoles may be observed but is not a consistent finding, and the mechanism of resistance in *A. niger* has not been fully elucidated, although it does not appear to be due to typical *cyp51* mutations or upregulation [32,33].

There is general concern over increasing azole resistance in IA pathogens associated with *cyp51* mutations [11,34], but data vary over time and between studies [24,25]. We saw no significant temporal increases in MIC values but did find variability between years. Analytical test factors, institution-specific clinical practices, and environmental variables may affect mold biology and MIC values [11,35]. Our increased baseline NWT rates for isavuconazole coincide with its use as prophylaxis and empiric or targeted therapy at our institution, yet MICs were not appreciably increased for posaconazole, which is also routinely utilized at our site for prophylaxis and therapy. An investigation into whether the MIC trends correlate with clinical exposure to antifungal agents can provide further insights for antifungal stewardship and interpretation of MIC data.

Echinocandins are known to cause morphological changes and growth arrest in *Aspergillus* spp.; therefore, determining MECs is recommended in order to assess their activity more accurately [36] MICs and MECs were identical for all of the echinocandins–organism combinations tested. In a transplant-associated surveillance network study, most *Aspergillus* spp. isolates collected between 2001 and 2006 showed MEC values not exceeding the ECVs defined for echinocandins [37]. Although the echinocandin agents generally had low MICs below ECVs for *Aspergillus* spp. in this study, we also found a selective increase in MICs/MECs to caspofungin among echinocandins in roughly half of the isolates identified as *A. nidulans* complex. This AST pattern has been described in other *Aspergillus* section *Nidulantes* species, particularly *A. spinulosporus*, but not with *A. nidulans* sensu stricto [38,39]. *A. spinulosporus* is a rare cause of invasive fungal infection in immunocompromised hosts and it is unknown if commercial MALDI-ToF systems would identify this closely related species as *A. nidulans*. We also visually observed an increasing trend in the MIC/MEC values of the echinocandins against some of the *Aspergillus* spp. (Figure 2). These observations were not statistically significant, which may be due to the multiplicative nature of MIC/MEC data and the limited number of collection years.

Non-*Aspergillus* mold isolates pose challenges in immunocompromised patients and in demonstrating elevated MICs to multiple antifungal drug classes. There are no CLSI interpretative standards or ECVs for these molds, though ECVs using the CLSI-based broth microdilution method have been described by other study groups [40,41]. For *Fusarium*, amphotericin B ECVs ranged from 4 to 8 µg/mL across species, similar to our data, but for other antifungal agents, ECVs varied much more greatly by species; therefore, we are unable to make meaningful comparisons [41]. For *Scedosporium/Lomentospora* spp., amphotericin B MICs are commonly increased, though in our dataset the MIC distribution (MIC_50_ = 8 µg/mL) is skewed higher than reported by others [42]. Elevated voriconazole MICs were also common in our isolates compared to prior studies, raising concerns of effective therapy options since voriconazole is a primary therapeutic agent for scedosporiosis [42,43]. For Zygomycetes, others have found variable but elevated MICs to isavuconazole simultaneously with favorable MICs to posaconazole [21,44], like the findings in the present study, and we additionally describe somewhat less-favorable MICs to itraconazole compared to posaconazole. Our findings for amphotericin B MIC distributions (ECV 97.5% of 2 µg/mL) were similar to previously reported ECVs of 2–4 µg/mL [40]. The clinical utility of AST performance for these non-*Aspergillus* molds remains to be proven, as clinical factors and other therapeutic modalities may be correlated more strongly with outcomes than MIC results from AST [45]. Additionally, it is hoped that newer antifungal agents in clinical trials, including olorofim and fosmanogepix, will soon become available for the treatment of IMIs caused by molds that are difficult to treat and that appear highly resistant in vitro [46,47].

Our collection of index mold isolates for antifungal susceptibility was tested at the time of care, minimizing selection bias and the effects of freeze–thaw and culture passages on MIC values. Our MIC testing and analysis was based on CLSI standards, recognizing that while there are also EUCAST standards for antifungal testing, several key differences exist between the two published AST methods [48]. In the absence of clinical data, we did not determine if all isolates were associated with IMIs and could not determine if elevated MIC values for certain agents were associated with patient parameters such as the cancer diagnosis or the prior use of antifungal agents.

This study offers epidemiological data to support the understanding of antifungal MICs across pathogenic molds in a high-risk cancer patient population commonly receiving antifungal therapy. Among the antifungal agents tested, posaconazole along with other echinocandins demonstrated consistently lower MICs across *Aspergillus* spp. Posaconazole and had a more favorable MIC distribution than isavuconazole and itraconazole against Zygomycetes. We further make note of elevated MICs for certain organism–antifungal agent combinations that should be monitored, including isavuconazole and *Aspergillus* and Zygomycetes, caspofungin and *A. nidulans*, and itraconazole and *A. niger*. While we describe these MIC trends, there are no clinical breakpoints to help interpret the applicability of these MIC values. Regardless, antifungal stewardship efforts are needed for the surveillance of unfavorable MICs and to help prevent the additional emergence of NWT strains. Although fungal antigen and molecular testing are widespread and important diagnostic tools, the microbiologic sampling and antifungal AST of recovered isolates remain vital to understand MIC patterns and emerging trends in vulnerable populations. Genotyping for *cyp51* and other alterations in *Aspergillus* spp. as well as clinical correlation studies can help further elucidate the significance of the elevated MICs and NWT populations that were observed.

## Figures and Tables

**Table 1:** List of identified *Aspergillus* spp. with respective MIC, MEC (for echinocandins only), ECV, and wild-type upper limits for different antifungal compounds tested.

Organisms	Agent	MIC/MEC_50_	MIC/MEC_90_	Mode	Range	ECV (97.5%)	wt-UL; (Mode + 2)	CLSI ECV
*Aspergillus flavus/oryzae* (*n* = 23)	5-FC	≥64	≥64	≥64	4–≥64		256	
	Ampho	1	2	1	0.25–4	4	4	4
	Anid	≤0.06	≤0.06	≤0.06	≤0.06		0.25	
	Casp	≤0.06	≤0.06	≤0.06	≤0.06		0.25	
	Fluc	≥128	≥128	≥128	32–≥128		512	
	ISAV	1	2	1	0.5–4	4	4	1
	Itra	0.12	0.25	0.12	≤0.06–1	0.5	0.5	1
	Keto	1	1	1	0.25–2	2	4	
	Mica	≤0.06	≤0.06	≤0.06	≤0.06		0.25	
	Pos	≤0.06	≤0.06	≤0.06	≤0.06–0.25		0.12	0.5
	Vori	1	2	1	0.5–2	2	4	2
*Aspergillus fumigatus* (*n* = 214)	5-FC	≥64	≥64	≥64	0.25–≥64		256	
	Ampho	1	2	2	0.25–4	4	8	2
	Anid	≤0.06	≤0.06	≤0.06	≤0.06–4		0.25	
	Casp	≤0.06	≤0.06	≤0.06	≤0.06–4		0.25	0.5
	Fluc	≥128	≥128	≥128	≤0.5–≥128		512	
	ISAV	1	2	1	0.25–≥16	2	4	1
	Itra	0.12	0.25	0.12	≤0.06–≥16	0.5	0.5	1
	Keto	4	8	4	≤0.06–≥16	8	16	
	Mica	≤0.06	≤0.06	≤0.06	≤0.06–≥8		0.25	
	Pos	≤0.06	≤0.06	≤0.06	≤0.06–0.5		0.25	
	Vori	0.5	1	0.5	0.12–≥16	2	2	1
*Aspergillus nidulans* (*n* = 24)	5-FC	≥64	≥64	≥64	32–≥64		256	
	Ampho	1	2	1	0.25–2	4	4	
	Anid	≤0.06	≤0.06	≤0.06	≤0.06–≥8		0.25	
	Casp	0.5	4	≤0.06	≤0.06–≥8		0.25	
	Fluc	≥128	≥128	≥128	32–≥128		512	
	ISAV	0.25	0.5	0.25	0.12–0.5	0.5	1	
	Itra	≤0.06	0.25	≤0.06	≤0.06–1	0.25	0.25	
	Keto	0.12	0.5	0.12	≤0.06–0.5	2	0.5	
	Mica	≤0.06	≤0.06	≤0.06	≤0.06–≥8		0.25	
	Pos	≤0.06	0.12	≤0.06	≤0.06–0.5		0.25	
	Vori	0.25	0.5	0.25	0.12–1	1	1	
*Aspergillus niger* complex (*n* = 92)	5-FC	32	≥64	≥64	1–≥64		256	
	Ampho	0.5	1	0.5	≤0.12–2		2	2
	Anid	≤0.06	≤0.06	≤0.06	≤0.06		0.25	
	Casp	≤0.06	≤0.06	≤0.06	≤0.06		0.25	0.25
	Fluc	≥128	≥128	≥128	≤0.5–≥128		512	
	ISAV	4	8	4	0.12–≥16	8	16	4
	Itra	0.5	≥16	0.5	≤0.06–≥16	1	2	4
	Keto	8	≥16	8	≤0.06–≥16	64	32	
	Mica	≤0.06	≤0.06	≤0.06	≤0.06		0.25	
	Pos	0.12	0.25	0.12	≤0.06–0.5	0.5	0.5	2
	Vori	2	2	2	0.06–4	4	8	2
*Aspergillus terreus* complex (*n* = 24)	5-FC	≥64	≥64	≥64	1–≥64		256	
	Ampho	2	4	4	0.5–≥8	8	16	4
	Anid	≤0.06	≤0.06	≤0.06	≤0.06		0.25	
	Casp	≤0.06	≤0.06	≤0.06	≤0.06–0.12		0.25	0.12
	Fluc	≥128	≥128	≥128	≤0.5–≥128		512	
	ISAV	0.5	1	0.5	0.12–2	2	2	1
	Itra	≤0.06	0.12	≤0.06	≤0.06–0.25		0.25	2
	Keto	0.5	1	0.5	0.12–2	2	2	
	Mica	≤0.06	≤0.06	≤0.06	≤0.06		0.25	
	Pos	≤0.06	≤0.06	≤0.06	≤0.06		0.25	1
	Vori	0.5	1	0.5	0.12–2	1	2	2
	5-FC	≥64	≥64	≥64	≥64		256	
*Aspergillus lentulus* (*n* = 9)	Ampho	4	≥8	4	1–≥8	8	16	
	Anid	≤0.06	≤0.06	≤0.06	≤0.06		0.25	
	Casp	≤0.06	≤0.06	≤0.06	≤0.06		0.25	
	Fluc	≥128	≥128	≥128	≤0.5–≥128		512	
	ISAV	4	8	4	2–8	8	16	
	Itra	0.25	2	0.25	0.25–2		1	
	Keto	8	≥16	8	2–≥16	8	32	
	Mica	≤0.06	≤0.06	≤0.06	≤0.06		0.25	
	Pos	≤0.06	0.5	≤0.06	≤0.06–0.5		0.25	
	Vori	2	4	2	0.25–4	8	8	

Abbreviations: 5-FC, Flucytosine; Ampho, Amphotericin B; Anid, Anidulafungin; Casp, Caspofungin; Fluc, Fluconazole; ISAV, Isavuconazole; Itra, Itraconazole; Keto, Ketoconazole; Mica, Micafungin; Pos, Posaconazole; Vori, Voriconazole. ECV (97.5%) notates ECVs determined by calculating the mean and the standard deviation of the modeled distribution and choosing the MIC that includes 97.5% of the wild-type population. Wt-UL, (Mode + 2) notes ECVs determined at 2 dilution steps above the modal MIC value.

**Table 2:** List of other identified molds with respective MIC, MEC (for echinocandins only), ECV, and wild-type upper limits for different antifungal compounds tested.

Organisms.	Agent	MIC/MEC_50_	MIC/MEC_90_	Mode	Range	ECV (97.5%)	wt-UL; (Mode + 2)
*Fusarium* spp. (*n* = 26)	5-FC	≥64	≥64	≥64	≥64		256
	Ampho	4	≥8	4	1–≥8	8	16
	Anid	≥8	≥8	≥8	≤0.06–≥8		32
	Casp	≥8	≥8	≥8	≤0.06–≥8		32
	Fluc	≥128	≥128	≥128	4–≥128		512
	ISAV	≥16	≥16	≥16	8–≥16		64
	Itra	≥16	≥16	≥16	≥16		64
	Keto	≥16	≥16	≥16	2–≥16		64
	Mica	≥8	≥8	≥8	≤0.06–≥8		32
	Pos	≥16	≥16	≥16	1–≥16		64
	Vori	≥16	≥16	≥16	0.5–≥16		64
*Scedosporium* spp. (*n* = 22)	5-FC	≥64	≥64	≥64	32–≥64		256
	Ampho	≥8	≥8	≥8	1–≥8		32
	Anid	4	≥8	4	≤0.06–≥8	4	16
	Casp	≥8	≥8	≥8	≤0.06–≥8		32
	Fluc	16	64	16	8–≥128	32	64
	ISAV	8	≥16	8	0.5–≥16	32	32
	Itra	4	≥16	≥16	0.5–≥16		64
	Keto	0.5	1	0.5	≤0.06–4	2	2
	Mica	≥8	≥8	≥8	≤0.06–≥8		32
	Pos	1	2	1	0.5–≥16	2	4
	Vori	1	4	1	0.12–≥16	4	4
*Lomentospora prolificans* (*n* = 17)	5-FC	≥64	≥64	≥64	≥64		256
	Ampho	≥8	≥8	≥8	4–≥8		32
	Anid	4	≥8	4	2–≥8	4	16
	Casp	≥8	≥8	≥8	2–≥8		32
	Fluc	≥128	≥128	≥128	16–≥128		512
	ISAV	≥16	≥16	≥16	4–≥16		64
	Itra	≥16	≥16	≥16	1–≥16		64
	Keto	≥16	≥16	≥16	0.5–≥16		64
	Mica	≥8	≥8	≥8	0.25–≥8		32
	Pos	≥16	≥16	≥16	0.5–≥16		64
	Vori	≥16	≥16	≥16	0.5–≥16		64
Zygomycetes (*n* = 49)	5-FC	≥64	≥64	≥64	32–≥64		256
	Ampho	1	2	1	≤0.12–≥8	2	4
	Anid	≥8	≥8	≥8	≤0.06–≥8		32
	Casp	≥8	≥8	≥8	≤0.06–≥8		32
	Fluc	≥128	≥128	≥128	16–≥128		512
	ISAV	4	≥16	2	0.5–≥16	8	8
	Itra	1	≥16	1	0.12–≥16	4	4
	Keto	1	4	1	0.25–≥16	8	4
	Mica	≥8	≥8	≥8	≤0.06–≥8		32
	Pos	0.5	32	0.5	≤0.06–≥16	2	2
	Vori	≥16	≥16	≥16	2–≥16		64

Abbreviations: 5-FC, Flucytosine; Ampho, Amphotericin B; Anid, Anidulafungin; Casp, Caspofungin; Fluc, Fluconazole; ISAV, Isavuconazole; Itra, Itraconazole; Keto, Ketoconazole; Mica, Micafungin; Pos, Posaconazole; Vori, Voriconazole. ECV (97.5%) notates ECVs determined by calculating the mean and the standard deviation of the modeled distribution and choosing the MIC that includes 97.5% of the wild-type population. Wt-UL, (Mode + 2) notes ECVs determined at 2 dilution steps above the modal MIC value. *Fusarium* spp. (*n* = 26): 10 *F. solani* complex 3 *F. proliferatum*, 3 *F. oxysporum* complex, 10 *Fusarium* species not further identified; *Scedosporium* spp. (*n* = 22): 7 *S. boydii*, 7 *S. apiospermum*, 4 *S. apiospermum/S. boydii* complex, 4 *Scedosporium* species not further identified; Zygomycetes (*n* = 49): 11 *Rhizopus microsporus* complex, 8 *Mucor* spp., 4 *Rhizopus arrhizus* complex, 4 *Rhizomucor* spp. 3 *Lichtheimia corymbifera*, 3 *Syncephalastrum* spp., 2 *Rhizopus miehei*, 2 *Rhizopus oryzae*, 1 *Rhizomucor pusillus*, 11 *Rhizopus* species not further identified.

**Table 3:** MIC and MEC (for echinocandins only) distributions for 11 antifungal agents of mold species presented in Table 1 and Table 2.

**5-FC (MIC; µg/mL)**	**≤0.06**	**0.12**	**0.25**	**0.5**	**1**	**2**	**4**	**8**	**16**	**32**	**≥64**	
*Aspergillus flavus/oryzae*							1			5	17	
*Aspergillus fumigatus*			1				1	3		14	194	
*Aspergillus nidulans*										1	23	
*Aspergillus niger* complex					1	1	8	17	18	20	27	
*Aspergillus terreus* complex					1				1	1	21	
*Aspergillus lentulus*											9	
*Fusarium* spp.											26	
*Scedosporium* spp.										1	19	
*Lomentospora prolificans*											17	
Zygomycetes										1	48	
**Ampho (MIC; µg/mL)**		**≤0.12**	**0.25**	**0.5**	**1**	**2**	**4**	**≥8**				
*Aspergillus flavus/oryzae*			1	5	8	8	1					
*Aspergillus fumigatus*			2	32	80	98	2					
*Aspergillus nidulans*			1	7	9	7						
*Aspergillus niger* complex		2	10	55	18	7						
*Aspergillus terreus* complex				1	4	7	10	2				
*Aspergillus lentulus*					1	3	4	1				
*Fusarium* spp.					4	6	10	6				
*Scedosporium* spp.					1		6	13				
*Lomentospora prolificans*							2	15				
Zygomycetes		3	3	9	25	6		3				
**Anid (MIC/MEC; µg/mL)**	**≤0.06**	**0.12**	**0.25**	**0.5**	**1**	**2**	**4**	**≥8**				
*Aspergillus flavus/oryzae*	23											
*Aspergillus fumigatus*	209	2			2		1					
*Aspergillus nidulans*	22							2				
*Aspergillus niger* complex	92											
*Aspergillus terreus* complex	24											
*Aspergillus lentulus*	9											
*Fusarium* spp.	2						1	23				
*Scedosporium* spp.	1					3	13	3				
*Lomentospora prolificans*						3	11	3				
Zygomycetes	1						12	36				
**Casp (MIC/MEC; µg/mL)**	**≤0.06**	**0.12**	**0.25**	**0.5**	**1**	**2**	**4**	**≥8**				
*Aspergillus flavus/oryzae*	23											
*Aspergillus fumigatus*	209	3	1				1					
*Aspergillus nidulans*	8		2	3	8		1	2				
*Aspergillus niger* complex	92											
*Aspergillus terreus* complex	23	1										
*Aspergillus lentulus*	9											
*Fusarium* spp.	2						1	23				
*Scedosporium* spp.	1			1	1	2	3	12				
*Lomentospora prolificans*					1	2		14				
Zygomycetes	1						1	47				
**Fluc (MIC; µg/mL)**				**≤0.5**	**1**	**2**	**4**	**8**	**16**	**32**	**64**	**≥128**
*Aspergillus flavus/oryzae*										2	7	14
*Aspergillus fumigatus*				5	2				1	3	15	188
*Aspergillus nidulans*										4	7	13
*Aspergillus niger* complex				2			1		1	5	7	76
*Aspergillus terreus* complex				1				1		2	6	14
*Aspergillus lentulus*				1							1	7
*Fusarium* spp.							1			1		24
*Scedosporium* spp.								6	7	3	3	1
*Lomentospora prolificans*									1			16
Zygomycetes									2	2	6	38
**ISAV (MIC; µg/mL)**	**≤0.06**	**0.12**	**0.25**	**0.5**	**1**	**2**	**4**	**8**	**≥16**			
*Aspergillus flavus/oryzae*				4	11	6	2					
*Aspergillus fumigatus*			3	45	124	32	5	2	3			
*Aspergillus nidulans*		7	12	5								
*Aspergillus niger* complex		1	1	1		22	43	20	4			
*Aspergillus terreus* complex		2	3	10	7	2						
*Aspergillus lentulus*						3	4	2				
*Fusarium* spp.								1	25			
*Scedosporium* spp.				1		3	1	9	6			
*Lomentospora prolificans*							1	2	14			
Zygomycetes				2	8	14	14	1	10			
**Itra (MIC; µg/mL)**	**≤0.06**	**0.12**	**0.25**	**0.5**	**1**	**2**	**4**	**8**	**≥16**			
*Aspergillus flavus/oryzae*	7	11	3		2							
*Aspergillus fumigatus*	34	104	57	14	2	1			2			
*Aspergillus nidulans*	13	8	2		1							
*Aspergillus niger* complex	2		18	31	11	2	1	1	26			
*Aspergillus terreus* complex	19	4	1									
*Aspergillus lentulus*			7		1	1						
*Fusarium* spp.									26			
*Scedosporium* spp.				1	5	3	2	2	7			
*Lomentospora prolificans*					1				16			
Zygomycetes		1		8	18	12	2	2	6			
**Keto (MIC; µg/mL)**	**≤0.06**	**0.12**	**0.25**	**0.5**	**1**	**2**	**4**	**8**	**≥16**			
*Aspergillus flavus/oryzae*			4	7	10	2						
*Aspergillus fumigatus*	3	2	1	8	20	57	96	23	4			
*Aspergillus nidulans*	7	9	3	5								
*Aspergillus niger* complex	2	2	1		5	9	22	31	20			
*Aspergillus terreus* complex		1	6	10	5	2						
*Aspergillus lentulus*						1	1	5	2			
*Fusarium* spp.						2		3	21			
*Scedosporium* spp.	2		2	9	5	1	1					
*Lomentospora prolificans*				1		1	2	4	9			
Zygomycetes			5	7	18	8	8	2	1			
**Mica (MIC/MEC; µg/mL)**	**≤0.06**	**0.12**	**0.25**	**0.5**	**1**	**2**	**4**	**≥8**				
*Aspergillus flavus/oryzae*	23											
*Aspergillus fumigatus*	211	1		1				1				
*Aspergillus nidulans*	22							2				
*Aspergillus niger* complex	92											
*Aspergillus terreus* complex	24											
*Aspergillus lentulus*	9											
*Fusarium* spp.	2						2	22				
*Scedosporium* spp.	1		2	1	4			12				
*Lomentospora prolificans*			1		2		3	11				
Zygomycetes	1						1	47				
**Pos (MIC; µg/mL)**	**≤0.06**	**0.12**	**0.25**	**0.5**	**1**	**2**	**4**	**8**	**≥16**			
*Aspergillus flavus/oryzae*	17	3	3									
*Aspergillus fumigatus*	197	11	4	2								
*Aspergillus nidulans*	17	5	1	1								
*Aspergillus niger* complex	28	46	15	3								
*Aspergillus terreus* complex	24											
*Aspergillus lentulus*	6	2		1								
*Fusarium* spp.					1	3			22			
*Scedosporium* spp.				2	11	5			2			
*Lomentospora prolificans*				1					16			
Zygomycetes	1		8	16	11	7		1	5			
**Vori (MIC; µg/mL)**	**≤0.06**	**0.12**	**0.25**	**0.5**	**1**	**2**	**4**	**8**	**≥16**			
*Aspergillus flavus/oryzae*				6	14	3						
*Aspergillus fumigatus*		1	30	109	56	13	3	1	1			
*Aspergillus nidulans*		3	10	9	2							
*Aspergillus niger* complex	1	1		3	28	51	8					
*Aspergillus terreus* complex		1	9	11	2	1						
*Aspergillus lentulus*			1			5	3					
*Fusarium* spp.				1		1	2	7	15			
*Scedosporium* spp.		1	1	5	6	4	2		1			
*Lomentospora prolificans*				1				1	14			
Zygomycetes						1	5	14	29			

Abbreviations: 5-FC, Flucytosine; Ampho, Amphotericin B; Anid, Anidulafungin; Casp, Caspofungin; Fluc, Fluconazole; ISAV, Isavuconazole; Itra, Itraconazole; Keto, Ketoconazole; Mica, Micafungin; Pos, Posaconazole; Vori, Voriconazole.

## Data Availability

The raw data supporting the conclusions of this article will be made available by the authors upon request.

## Supplementary Materials

The following supporting information can be downloaded at: https://www.mdpi.com/article/10.3390/jof11070518/s1: Figure S1: Number of isolated *Aspergillus* spp. and its trends over the years. Figure S2: Distribution of isolated molds in various specimens from cancer patients. Table S1: Total counts and percentage of different mold and yeast species isolated from cancer patients.

## Abbreviations

The following abbreviations are used in this manuscript:

5-FC	Flucytosine
Ampho	Amphotericin B
Anid	Anidulafungin
Aspfla	*A. flavus*
Aspfum	*A. fumigatus*
Aspnid	*A. nidulans*
Aspnig	*A. niger*
Aspter	*A. terreus*
AST	Antimicrobial susceptibility testing
Casp	Caspofungin
ECOFFs/ECVs	Epidemiological cutoff values
Fluc	Fluconazole
IA	Invasive aspergillosis
IMI	Invasive mold infections
ISAV	Isavuconazole
Itra	Itraconazole
Keto	Ketoconazole
MEC	Minimal effective concentrations
MIC	Minimal inhibitory concentrations
Mica	Micafungin
NWT	Non-wild-type
Pos	Posaconazole
Vori	Voriconazole
wt-UL	Upper limit of the wild-type population
