# Supplementary material for: Antifungal Minimal Inhibitory Concentrations of Mold Isolates from Patients with Cancer; Single-Center Experience, 2018–2023
Source: J Fungi (Basel). 2025 Jul 12;11(7):518. doi: 10.3390/jof11070518 (PMC12298419; doi:10.3390/jof11070518)
Supplement: Supplementary file 1 [file jof-11-00518-s001.zip › jof-3722918-supplementary.pdf]

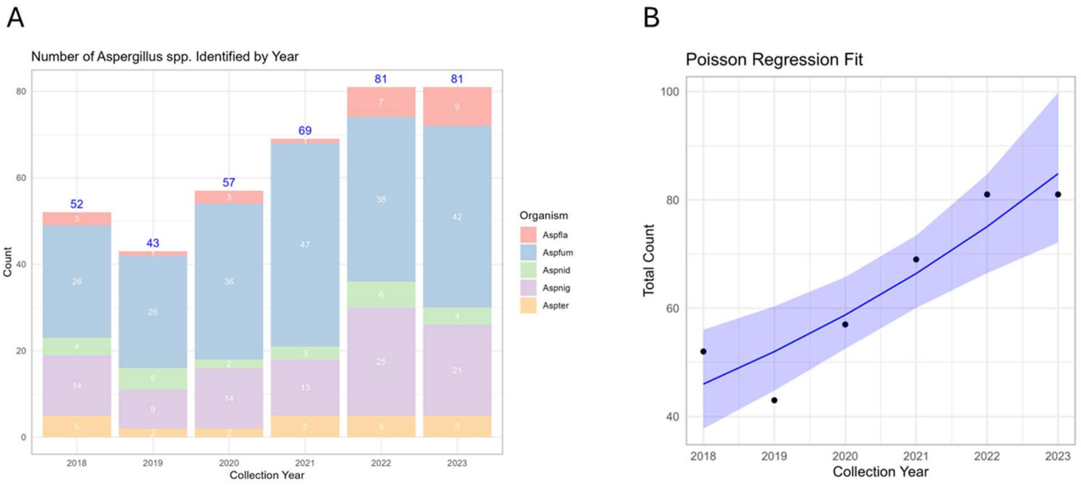

Figure S1: **Number of isolated *Aspergillus* spp. and its trends over the years**. The yearly distribution of the five most abundant *Aspergillus* spp. identified from clinical isolates between 2018 to 2023 (A). The shaded blue region represents the 95% confidence band around the fitted trend, indication that the model consistently predicts higher isolate counts in later years compared to earlier years (B). The fitted Poisson regression line represents a statistically significant upward trend for the observed isolate counts over the years (p = 0.000046).

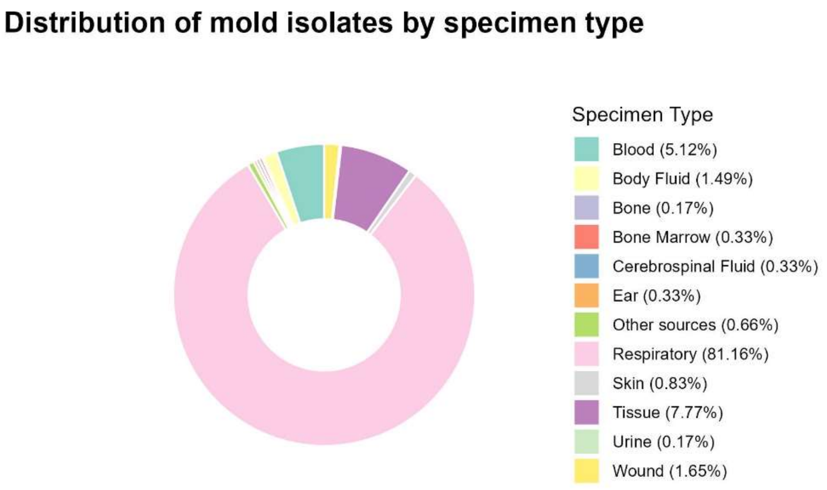

Figure S2: Distribution of isolated molds in various specimens from cancer patients. The distributio of mold species are presented as percentages.

Table S1: Total counts and percentage of different mold and yeast species isolated from cancer patients. If multiple organisms are listed per row, the count refers to number of each organism.

Organisms	Count	Percentage (%)
Aspergillus fumigatus	215	20.97
Candida albicans	109	10.63
Candida glabrata	105	10.24
Aspergillus niger	96	9.36
Candida krusei	56	5.46
Zygomycetes	49	4.78
Candida tropicalis	32	3.12
Fusarium spp.	27	2.63
Aspergillus flavus, Aspergillus nidulans, Aspergillus terreus,	24	2.34
Scedosporium apiospermum, Scedosporium boydii	22	2.14
Candida lusitaniae, Candida parapsilosis	18	1.75
Lomenstospora prolificans	17	1.65
Candida guilliermondii	14	1.36
Aspergillus spp., *Trichosporon asahii*	11	1.07
Aspergillus lentulus, Cryptococcus neoformans, Penicillium spp.	9	0.87
Saccharomyces cerevisiae	8	0.78
Alternaria alternata, Candida kefyr, Candida orthopsilosis	7	0.68
Candida auris, Coccidioides immitis, Other spp.	6	0.58
Paecilomyces spp.	5	0.48
Aspergillus versicolor, Cladosporium species, Coprinellus micaceus	4	0.39
Aspergillus glaucus, Candida famata	3	0.29
Acremonium spp., *Aspergillus ustus, Candida dubliniensis, Candida inconspicua, Lomentospora prolificans, Ochroconis* spp., *Purpureocillium lilacinum, Rhodotorula mucilaginosa, Saprochaete capitata, Scopulariopsis* spp.	2	0.19
Alternaria spp., *Aspergillus ochraceus, Beauveria bassiana, Bipolaris* spp., *Candida freyschussii, Candida lipolytica, Candida magnoliae, Candida metapsilosis, Cephalotheca* spp., *Chaetomium* spp., *Cladophialophora bantiana, Cryptococcus albidus, Cryptococcus gattii, Cryptococcus uzbekistanensis, Curvularia lunata, Curvularia spicifera, Debaryomyces hansenii, Dematiaceous* molds, *Epicoccum* spp., *Exophiala dermatitidis, Fomitopsis palustris, Gibellulopsis nigrescens, Humicola* spp., *Microsporum canis, Microascus murinus, Microascus* spp., *Myceliophthora* spp., *Nigrospora oryzae, Penicillium digitatum, Phanerochaete* spp., *Phaeoacremonium parasiticum, Rasamsonia argillacea, Schizophyllum* spp., *Scopulariopsis brevicaulis, Scytalidium lignicola, Scytalidium* spp., *Trichosporon* spp., *Ulocladium* spp.	1	0.09
